# Type I Angiotensin II Receptor Blockade Reduces Uremia‐Induced Deterioration of Bone Material Properties

**DOI:** 10.1002/jbmr.4159

**Published:** 2020-10-02

**Authors:** Takuya Wakamatsu, Yoshiko Iwasaki, Suguru Yamamoto, Koji Matsuo, Shin Goto, Ichiei Narita, Junichiro J Kazama, Kennichi Tanaka, Akemi Ito, Ryosuke Ozasa, Takayoshi Nakano, Chisato Miyakoshi, Yoshihiro Onishi, Shingo Fukuma, Shunichi Fukuhara, Hideyuki Yamato, Masafumi Fukagawa, Tadao Akizawa

**Affiliations:** ^1^ Division of Clinical Nephrology and Rheumatology Niigata University Graduate School of Medical and Dental Sciences Niigata Japan; ^2^ Department of Health Sciences Oita University of Nursing and Health Sciences Oita Japan; ^3^ Department of Nephrology and Hypertension Fukushima Medical University Fukushima Japan; ^4^ Ito Bone Histomorphometry Institute Niigata Japan; ^5^ Division of Materials and Manufacturing Science, Graduate School of Engineering Osaka University Suita Japan; ^6^ Department of Healthcare Epidemiology, School of Public Health in the Graduate School of Medicine Kyoto University Kyoto Japan; ^7^ Department of Pediatrics Kobe City Medical Center General Hospital Kobe Japan; ^8^ Institute for Health Outcomes and Process Evaluation Research (iHope International) Kyoto Japan; ^9^ The Keihanshin Consortium for Fostering the Next Generation of Global Leaders in Research (K‐CONNEX) Kyoto Japan; ^10^ Division of Nephrology, Endocrinology, and Metabolism Tokai University School of Medicine Isehara Japan; ^11^ Division of Nephrology, Department of Medicine Showa University School of Medicine Tokyo Japan

**Keywords:** BONE MATERIAL PROPERTIES, CHRONIC KIDNEY DISEASE, OLMESARTAN, RENIN‐ANGIOTENSIN‐ALDOSTERONE SYSTEM

## Abstract

Chronic kidney disease (CKD) is associated with a high incidence of fractures. However, the pathophysiology of this disease is not fully understood, and limited therapeutic interventions are available. This study aimed to determine the impact of type 1 angiotensin II receptor blockade (AT‐1RB) on preventing CKD‐related fragility fractures and elucidate its pharmacological mechanisms. AT‐1RB use was associated with a lower risk of hospitalization due to fractures in 3276 patients undergoing maintenance hemodialysis. In nephrectomized rats, administration of olmesartan suppressed osteocyte apoptosis, skeletal pentosidine accumulation, and apatite disorientation, and partially inhibited the progression of the bone elastic mechanical properties, while the bone mass was unchanged. Olmesartan suppressed angiotensin II‐dependent oxidation stress and apoptosis in primary cultured osteocytes in vitro. In conclusion, angiotensin II‐dependent intraskeletal oxidation stress deteriorated the bone elastic mechanical properties by promoting osteocyte apoptosis and pentosidine accumulation. Thus, AT‐1RB contributes to the underlying pathogenesis of abnormal bone quality in the setting of CKD, possibly by oxidative stress. © 2020 The Authors. *Journal of Bone and Mineral Research* published by Wiley Periodicals LLC on behalf of American Society for Bone and Mineral Research (ASBMR).

## Introduction

Chronic kidney disease (CKD) induces systemic mineral metabolism abnormalities, which constitute a condition termed CKD‐mineral and bone disorder (CKD‐MBD).^(^ ^1^ ^)^ Although many nephrologists believe that parathyroid dysfunction is the main cause of fragility fractures in CKD patients, clinical studies have failed to provide evidence to make a consensus on this issue.^(^ ^2^, ^3^, ^4^, ^5^ ^)^ Some studies even found no significant association between parathyroid function and fracture risk,^(^ ^6^, ^7^ ^)^ while there seems to be no argument regarding the elevated risk of fractures in CKD patients.^(^ ^8^, ^9^, ^10^, ^11^, ^12^ ^)^

Experimental kidney damage has been shown to induce abnormal bone mechanical properties without reducing the bone mass in animal models.^(^ ^13^, ^14^, ^15^ ^)^ The severity of these abnormalities was associated with the level of uremic toxins^(^ ^13^, ^14^ ^)^ but not with the parathyroid hormone (PTH) level.^(^ ^14^ ^)^

Osteoblasts express type 1 angiotensin II receptors (AT‐1R),^(^ ^16^ ^)^ which assist osteoclastogenesis by promoting the receptor activator for nuclear factor‐κB ligand.^(^ ^17^ ^)^ Therefore, an AT‐1R blockade (AT‐1RB) may have the potential to suppress osteoclastic bone resorption. A previous observational clinical study showed that the administration of renin‐angiotensin‐aldosterone system inhibitors (RASi), including AT1‐RB, was associated with an approximately 30% lower risk of hospital admission due to any fracture among patients with secondary hyperparathyroidism undergoing hemodialysis.^(^ ^18^ ^)^ The effect of RASi on preventing fractures in patients with CKD seemed more evident than that in the general population;^(^ ^19^ ^)^ however, the reason behind this finding remains unknown.

In this study, we aimed to determine the impact of AT‐1RB administration on preventing fragility fractures in patients undergoing hemodialysis and to elucidate the pharmacophysiological mechanisms underlying its preventive effect against bone fragility, with a special focus on the bone material properties.

## Materials and Methods

### Clinical study

#### Study design

The MBD‐5D study was a multicenter, prospective, observational study designed to follow patients with secondary hyperparathyroidism receiving maintenance hemodialysis for 3 years. Details of the study design have been reported previously.^(^ ^20^ ^)^ In short, the MBD‐5D study followed 8229 patients from January 2008 to January 2011. Forty percent (*n* = 3276) of patients were randomly selected as a subcohort, and their data were used in this analysis. The mean observation period was 2.74 years; all study procedures were conducted in accordance with the Declaration of Helsinki. Because this was an observational study of anonymized data collected during routine practice, informed consent from subjects was not mandatory according to the ethical guidelines for epidemiological research in Japan. The study protocol and the informed consent waiver were approved by the central ethics committee at Kobe University (no. 754).

#### Inclusion and exclusion criteria

Patients undergoing maintenance hemodialysis at a participating facility as of January 1, 2008, and who satisfied any of the inclusion criteria (intact PTH [iPTH] level of ≥180 pg/mL or treatment with intravenous vitamin D receptor activator or oral active vitamin D receptor activator) were eligible to participate. Patients undergoing hemodialysis for <3 months were excluded.

#### Outcomes and exposure

The outcome of interest in this study was the time to hospitalization due to any type of fracture. The period from the start of observation to the occurrence of the first event was recorded. Hospitalization for a fracture at any site was designated as “any fracture”; if medical record review indicated a hip fracture, this subset was designated as “hip fracture.” A participant was considered to be censored when time‐to‐event information was not available because of loss to follow‐up or occurring of the initial event. The exposure of interest was the use of AT1‐RB at study onset. All data were obtained from each patient's medical record.

#### Statistical processing

The distributions of continuous and categorical variables are presented as median (interquartile range) and percentages, respectively. The baseline characteristics of patients were compared between those hospitalized due to any fracture and those not hospitalized, employing a *t* test or chi‐square test for continuous and categorial variables, respectively.

To investigate the association between factors including the use of AT1‐RB and incidence of hospitalization due to any fracture, and that due to hip fracture, multivariable‐adjusted Cox proportional hazard models were used to estimate the hazard ratios (HRs) and their 95% confidence intervals (CIs). The following covariates were included in the model: age; sex; body mass index; cause of end‐stage kidney disease; diabetes mellitus; history of cardiovascular disease; history of parathyroidectomy; duration of dialysis; urea removal during a hemodialysis session (Kt/V); serum levels of albumin, calcium, phosphorus, iPTH, and alkaline phosphatase (ALP); use of AT1‐RB; and use of other antihypertensives, vitamin D receptor activators, and phosphate binders. CKD‐MBD levels were categorized as follows: calcium, <8.4 g/dL, 8.4 to 10.0 g/dL, and >10 g/dL; phosphorus, <3.5 g/dL, 3.5 to 6.0 g/dL, and >6.0 g/dL; iPTH, <60 pg/mL, >60 to <240 pg/mL, >240 to <500 pg/mL, and > 500 pg/mL;^(^ ^21^ ^)^ and ALP, four categories according to quartiles.

All analyses were performed using SAS 9.2 (SAS Institute, Cary, NC, USA) and differences with *p* < 0.05 were considered significant.

### Experimental studies

#### Materials

Alfa‐minimal essential medium (α‐MEM), penicillin 100 units/mL, and streptomycin 100 μg/mL were purchased from Life Technologies (Grand Island, NY, USA). Fetal bovine serum (FBS) and calf serum (CS) were purchased from HyClone Laboratories (Logan, UT, USA). Olmesartan was kindly supplied by Daiichi‐Sankyo Pharmaceutical Co. Ltd (Tokyo, Japan). Hydralazine and angiotensin II were purchased from Sigma (St. Louis, MO, USA). The fluorescent probe 2′,7′‐dihydro‐fluorescein diacetate (Molecular Probes, Eugene, OR, USA) was purchased from Invitrogen (Shibaura, Tokyo, Japan).

### In vivo study

#### Animal care

This study was approved by the Institutional Review Board of the Niigata University Hospital, Niigata, Japan (#269‐1). Sprague–Dawley male rats were maintained at a room temperature of 21°C to 24°C, humidity of 30% to 35%, and light and dark cycles of 12 hours each (8:00 a.m. to 8:00 p.m. in light and 8:00 p.m. to 8:00 a.m. in the dark). Until the age of 13 weeks, the rats were kept on a standard diet (MF; 1.07% calcium, 0.83% phosphorus) (Oriental Yeast Co. Ltd., Tokyo, Japan); at the age of 14 weeks, the rats were started on a special formula diet (CE‐2; 2.0% calcium, 1.0% phosphorus) (CLEA Japan, Tokyo, Japan). Rats were allowed *ad libitum* access to food and tap water. At the age of 14 to 15 weeks, the rats underwent a sham operation (group C) or a 5/6 subtotal nephrectomy under general anesthesia with isoflurane. More precisely, they underwent a total right nephrectomy and a 2/3 left nephrectomy with a 1‐week interval. At the age of 21 weeks, 5/6 nephrectomized rats were distributed into three groups, the NO, NH, and N groups, and were administered olmesartan (RNH‐6270) (3 mg/kg/d), hydralazine hydrochloride (10 mg/kg/d), and saline, respectively, using Alzet osmotic mini pump model 2ML2 (Durect, Cupertino, CA, USA). Olmesartan and hydralazine hydrochloride (model 2ML2) pumps were exchanged every 2 weeks. The body weight, blood pressure, and pulse rate of the rats were recorded at 26 weeks of age. Systolic, diastolic, and mean blood pressure and pulse rates were measured using the tail‐cuff method with heating, as previously described.^(^ ^22^ ^)^ Briefly, rats were preheated in a chamber at 39°C for 10 minutes. A cuff with a pneumatic pulse sensor was attached to the tail (BP‐98A‐L; Softron Beijing Incorporated, Beijing, China). Blood pressure and pulse rate values were averaged from at least four consecutive readings in each rat. Rats at the age of 27 weeks were deeply anesthetized with isoflurane and euthanized to collect femurs and blood for biochemical analysis.

#### Measurement of biochemical parameters:

Blood was centrifuged for 20 minutes at 2014 g and serum samples were used to determine urea nitrogen, creatinine, calcium, and inorganic phosphorus levels (SRL, Inc., Tokyo, Japan). Plasma was separated using EDTA‐2Na and centrifuged for 15 minutes at 1600*g*; the supernatant was used for measurement of plasma intact PTH concentration using an ELISA kit (Immutopics, San Clemente, CA, USA) according to the manufacturer's instructions.

#### Bone mineral densitometry (BMD)

The BMD values of the right femur were measured by dual‐energy X‐ray absorptiometry (DXA; Aloka DCS‐600R; Aloka Co., Tokyo, Japan).

#### Bone histomorphometry

The left femur of each rat was collected and fixed in 70% ethanol. The fixed femurs were stained with Villanueva Bone Stain Solution for 6 days en bloc and thereafter embedded into a methyl methacrylate resin. The histomorphometry of cancellous bone fields at 320× magnification in each specimen was analyzed. The results were expressed according to previously described methods.^(^ ^23^, ^24^ ^)^

#### Dynamic mechanical analysis (DMA)

DMA was performed as described previously.^(^ ^13^, ^14^, ^15^ ^)^ Briefly, each right femur that was used for BMD measurement was placed in a DMA device (DMA 7e; PerkinElmer, Norwalk, CT, USA), and the baseline viscoelasticity was measured in a 0.9% saline solution at 37°C by an oscillatory test using a 3‐point bending configuration. Before DMA, the thickness and width were measured at the center of each femur. The femur sample was then placed in the DMA device to determine the standard DMA profile. Scanning frequencies ranged from 0.1 to 25 Hz (in 0.2 Hz increments). The test was conducted under displacement control. The storage modulus E1 (obtained from dynamic testing, equivalent to Young's modulus) was measured for each sample.^(^ ^15^ ^)^

#### Confocal Raman spectroscopic measurements

Each femur sample used for DMA measurement was cut at the center of the diaphysis. Each cross‐sectional surface was polished and smoothed. A Nicolet Almega XR Dispersive Raman microscope system equipped with the OMNIC Atlus TM imaging software (Thermo Fisher Scientific, Auburn, AL, USA) was used to examine the composition and relative amounts of minerals and bone matrix, as previously described.^(^ ^13^, ^14^, ^15^ ^)^ Three cross‐sectional surfaces (anterior, posterior, and interior of the femur) as previously described^(^ ^16^ ^)^ were irradiated using a high‐brightness, low‐intensity laser operating at 780 nm. Raman spectral images were acquired and the average values of the three points were calculated. Each parameter was calculated as follows: the relative bone mineral amount was estimated by the v1 phosphate stretching vibration at 950 to 964 cm^−1^. In the same way, the relative bone matrix amount was estimated by amide III (1242 to 1269 cm^−1^). The carbonate/phosphate ratio was calculated as the ratio of the band area of type B carbonate substitution (1065 to 1070 cm^−1^) to v1 phosphate substitution (950 to 964 cm^−1^). Collagen maturity was calculated as the ratio of 1660 cm^−1^ (mature collagen) to 1690 cm^−1^ (immature collagen). The amount of pentosidine was estimated as the sum of the band areas that peaked at 1305 cm^−1^ and 1362 cm^−1^. For each sample, three averaged Raman spectral images were acquired in the middle of the anterior cortical bone from the femoral cross‐sectional surface (each image was acquired using 10 times measurements) and the mean values were calculated.

### In vitro study

#### Osteocytes preparation

Primary osteocytes were isolated from mouse hindlimb bones, according to the combined methods of Mikuni‐Takagaki and colleagues^(^ ^24^ ^)^ and Stern and colleagues^(^ ^25^ ^)^ with some modifications. Briefly, the hindlimb long bones (femur and tibia) were aseptically dissected from 10‐week‐old Institute of Cancer Research mice (SRL, Hamamatsu, Japan). The bones were minced into 1 mm pieces and serial digested with 1 mg/mL collagenase (Wako, Osaka, Japan) five times for 20 minutes each in α‐MEM at 37°C. The cells were then collected through a 100‐μm nylon cell strainer. Cells of the first and second fractions were discarded because these fractions contain abundant fibroblastic cells. Cells from fractions 3 to 5 were pooled as osteoblast‐rich fractions. Residual bone pieces were treated with 4 mM EGTA in Ca^2+^‐free, Mg^2+^‐free Hanks' balanced salt solution for 15 minutes (fraction 6) and 1 mg/mL collagenase for 20 minutes (fraction 7) to collect the cells. These digestions were repeated until fraction 11 was obtained. Fractions 6 and 7 (fraction 6/7), 8 and 9 (fraction 8/9), and 10 and 11 (fraction 10/11) were combined because of the low cell number. We performed a real‐time polymerase chain reaction (PCR) analysis using RNA extracted from 4‐day‐cultured cells in fractions 3 to 10/11 to examine the expressions of osteoblast and osteocyte marker genes (Supplemental Fig. S1). Keratocan was the marker gene of osteoblasts,^(^ ^26^ ^)^ while SOST and FGF23 were the marker genes of osteocytes. Keratocan was strongly expressed in fraction 3, whereas the opposite was found in fractions 8/9 and 10/11. However, the expression of SOST was higher in fractions 6/7, 8/9, and 10/11. FGF23 gene expression was higher in fractions 8/9 and 10/11 than in fraction 3, which also had the lowest SOST expression. These results were in agreement with those of a previous study,^(^ ^21^ ^)^ and we confirmed fractions 3 to 5 as osteoblast‐rich and fractions 6/7, 8/9, and 10/11 as osteocyte‐rich. Osteocytic cells were cultured on type I collagen‐coated plates (Becton Dicknson, Franklin Lakes, NJ, USA) at a seeding density of 2 × 10^6^ cells/9 cm^2^ in α‐MEM supplemented with 5% FBS and 5% CS, penicillin 100 units/mL, and streptomycin 100 μg/mL.

**Fig 1:**
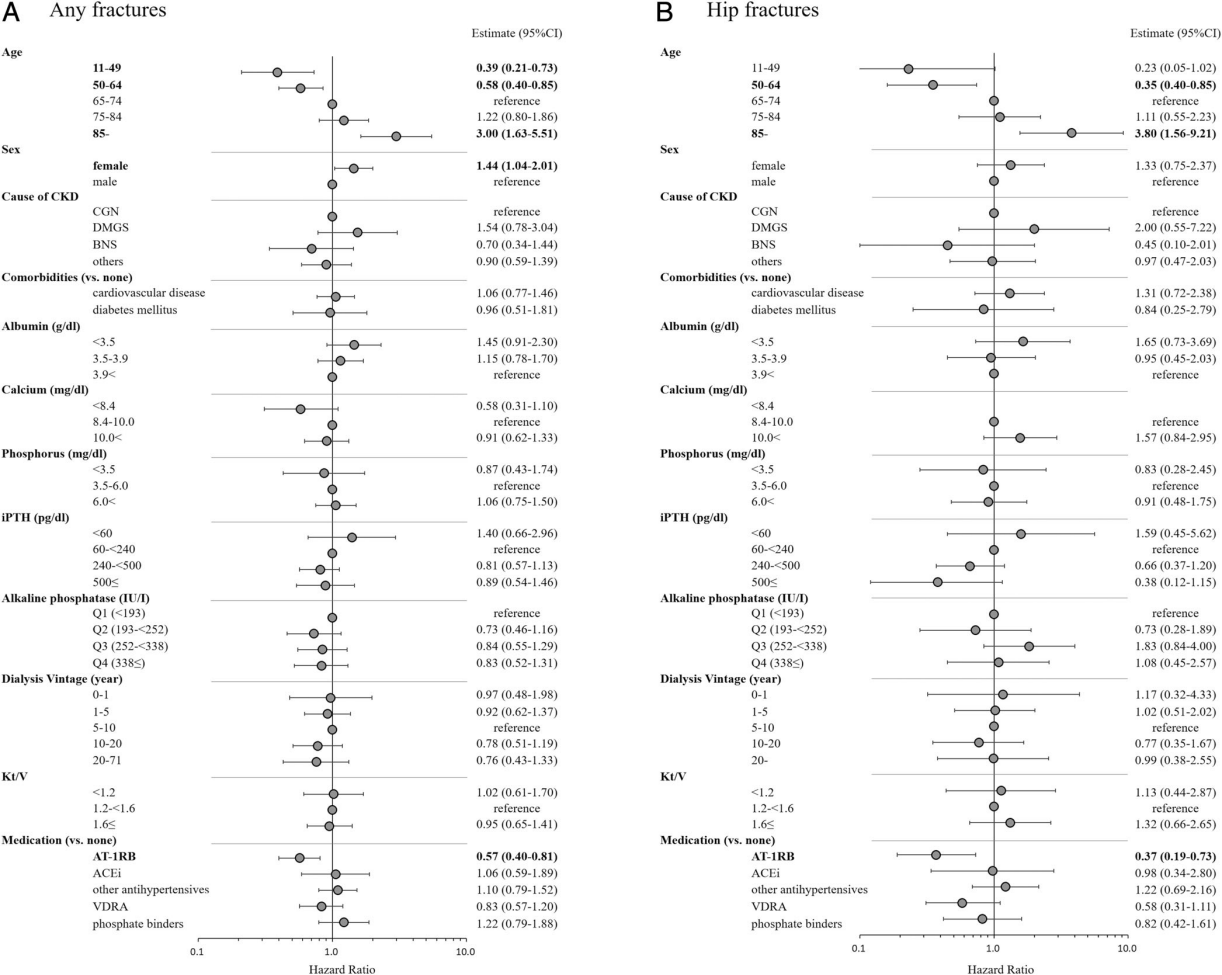
Relationship between time to hospitalization due to fracture and clinical factors. Association between CKD‐MBD‐related clinical parameters and the incidence of hospitalization due to (*A*) any fracture and (*B*) hip fracture. Multivariate‐adjusted Cox proportional hazard models were used to estimate hazard ratios (HRs) and their 95% confidence intervals (CIs). In the multivariate analysis, the following covariates were adjusted for: age, sex, body mass index, causes of chronic kidney disease (CKD), smoking, history of parathyroidectomy, duration of dialysis, serum levels of albumin, calcium, phosphorus, intact parathyroid hormone (iPTH), alkaline phosphatase, blood hemoglobin, prescriptions of type I angiotensin II receptor blockade (AT‐1RB), angiotensin converting enzyme inhibitor (ACEi), vitamin D receptor activators (VDRA), and phosphate binders.

#### RNA isolation, cDNA synthesis, and PCR analysis

To confirm the successful isolation of osteoblasts and osteocytes from mouse hindlimb long bones, we analyzed the gene expression in cultured cells. Five days post‐isolation, the total RNA was isolated from plated cells using the RNeasy Mini Kit (Qiagen, Tokyo, Japan) according to the manufacturer's instructions. The total RNA (1 μg) was used as the template for cDNA synthesis with a 20‐μL volume using an RT‐PCR kit (Invitrogen, Carlsbad, CA, USA) according to the manufacturer's instructions. Real‐time PCR was performed using 1 μL of cDNA with a 20‐reaction volume using StepOne Plus (Applied Biosystems, Waltham, MA, USA). We used SYBR Green chemistry to determine the mRNA levels of SOST, FGF23, keratocan, and a housekeeping gene, beta‐actin. The double‐stranded DNA‐specific dye SYBR Green I was incorporated into the PCR buffer provided in the SYBR Green Real‐Time PCR Master Mix (Toyobo Co. Ltd., Tokyo, Japan) to enable quantitative detection of products. The PCR conditions were 95°C for 30 seconds, 40 denaturation cycles at 94°C for 5 seconds, and annealing and extension at 60°C for 30 seconds. The primer sequences used are shown in Supplemental Table S1. Beta‐actin was used to normalize the differences in reverse transcription efficiency. We also assessed *AT‐1R* gene expression and determined the effect of AT‐1RB on angiotensin II‐induced NADPH oxidase gene expression.

#### Assessment of reactive oxygen species (ROS) production

Intracellular ROS was quantified as previously described.^(^ ^27^, ^28^ ^)^ Primary osteocytic cells were seeded into a 96‐well microplate at a density of 2 × 10^4^ cells/well. After incubation for 4 days, the osteocytes were washed with D‐PBS. Then 100 μL of D‐PBS containing 5 mM _D_‐glucose and 20 μM DCFH‐DA together with various concentrations (0 to 1000 nM) of angiotensin II were added to the wells. Olmesartan (10 mM), hydralazine (30 mM), or vehicle was simultaneously added to the wells. Immediately after the addition and after incubation at 37°C for 120 minutes, the fluorescence intensity was analyzed using a fluorescence plate reader (Spectromax Gemini XS, Molecular Devices, Sunnyvale, CA, USA) at excitation/emission wavelengths of 485/535 nm. Data were expressed as percent increase in fluorescence intensity compared with the untreated control. ROS production was also determined in the presence of 10 μM olmesartan, 30 μM hydralazine, 2.5 mM N‐acetyl cysteine, or 0.5 μM diphenyleneiodonium.

#### Measurement of cell viability

Cell viability was evaluated using an MTT (3‐[4,5‐dimethylthiazol‐2‐yl]‐2,5‐diphenyl‐tetrazolium bromide) assay (Dojin Chemicals, Kumamoto, Japan) as described previously.^(^ ^26^ ^)^ Primary osteocytic cells (2 × 10^4^ cells/well) were inoculated into 96‐well microplates. After culturing for 4 days, various concentrations (0 to 1000 nM) of angiotensin II were added and the cells were incubated at 37°C for 24 to 96 hours. Then the cells were lysed with isopropanol/HCl solution, and the absorbance of each well was measured at 570 nm with absorbance at 630 nm as reference, using a microplate reader (SpectraFluor; Tecan, Grodig, Austria). We also examined the effect of olmesartan, hydralazine, and antioxidants on cell viability with 1000 nM of angiotensin II.

#### Measurement of DNA fragmentation

For the assessment of apoptosis‐induced DNA fragmentation, osteocytic cells were seeded in a 96‐well microplate at a density of 2 × 10^4^ cells/well and incubated at 37°C for 4 days. Various concentrations (0 to 1000 nM) of angiotensin II were added and the cells were incubated for a further 48 hours. DNA fragmentation was assessed using the Cellular DNA Fragmentation ELISA Kit (Roche Diagnostics, Basel, Switzerland) according to the manufacturer's instructions.

#### Statistics

All data were expressed as mean ± standard deviation. The data were statistically analyzed by the Mann–Whitney *U* test. Analyses were performed using SAS 9.2 and differences with *p* < 0.05 were considered significant.

## Results

### The association between AT‐1RB use and fractures in patients undergoing hemodialysis

During the observation period, 178 (5.4%) of the 3276 patients were hospitalized due to fracture, of which 58 were due to a hip fracture. The baseline characteristics of the participants are shown in Table 1. All baseline covariates included in the regression model were retrospectively collected from medical records, and there were no missing data.

**Table 1:** Characteristics of the Analysis Sample

		Hospitalization due to any fracture		*p* Value
	All	No	Yes	
Variable	*N* = 3276	*n* = 3098	*n* = 178	
Demographic and clinical characteristics				
Age (years)	63 (54–71)	62 (54–71)	69 (60–76)	<0.001
Sex (female)	38.5%	37.8%	50.6%	0.001
Body mass index (kg/m^2^)	20.9 (19.0–23.3)	20.9 (19.1–23.3)	20.2 (18.3–23.1)	0.031
Cause of ESKD				
Glomerulonephritis	44.9%	45.0%	43.3%	
Diabetic nephropathy	24.2%	23.8%	31.5%	
Nephrosclerosis	5.8%	5.9%	5.1%	
Others	25.2%	25.5%	20.3%	
Comorbid conditions				
Diabetes mellitus	31.3%	31.0%	37.6%	0.062
Cardiovascular disease	60.0%	59.7%	65.2%	0.146
Dialysis				
Vintage (years)	8.3 (3.8–14.3)	8.3 (3.8–14.3)	7.6 (2.9–13.7)	0.689
Kt/V	1.41 (1.23–1.58)	1.41 (1.23–1.57)	1.45 (1.25–1.61)	0.232
Laboratory data				
Albumin (g/dL)	3.8 (3.5–4.0)	3.8 (3.5–4.0)	3.7 (3.4–3.9)	0.671
Calcium (mg/dL)	9.4 (8.9–10.1)	9.4 (8.9–10.1)	9.4 (8.8–10.0)	0.856
Phosphate (mg/dL)	5.5 (4.6–6.3)	5.5 (4.6–6.3)	5.3 (4.5–6.2)	0.275
Intact PTH (pg/mL)	265 (195–392)	267 (196–394)	242 (192–338)	0.926
Alkaline phosphatase (IU/L)	252 (197–330)	252 (197–329)	254 (196–353)	0.188
Medication				
AT‐1RB	35.4%	36.0%	24.7%	0.002
Calcium channel blocker	43.7%	43.8%	41.6%	0.560
VDRA	48.7%	48.8%	46.6%	0.567
Phosphate binder	85.3%	85.4%	84.8%	0.851

ESKD = end‐stage kidney disease; PTH = parathyroid hormone; AT‐1RB = angiotensin II type I receptor blockade; VDRA = vitamin D receptor agonist.

The median age of patients was 63 (54 to 71) years. More than 60% were male. The mean age of patients hospitalized due to fracture was higher than that of those who were not (68.1 years versus 61.6 years, *p* < 0.001). Among those hospitalized for fracture, the proportion of female patients was 50.6%, which was higher than that in patients not hospitalized (37.8%, *p =* 0.001). Additionally, the body mass index was lower in patients with fractures than in those without fractures.

AT‐1RB use was lower in patients hospitalized due to fractures than in those without fractures (24.7% versus 36.0%, *p =* 0.002). The results of Cox regression analyses with respect to the patients' background data are shown in Fig. 1. AT‐1RB use also showed an association with a lower risk of hospitalization due to any fracture (hazard ratio [HR] = 0.57, 95% confidence interval [CI] 0.40–0.81, *p* < 0.01) (Fig. 1*A*) and hip fractures (HR = 0.37, 95% CI 0.19–0.73, *p* < 0.01) (Fig. 1*B*), while the use of vitamin D receptor activators (VDRA) and phosphate binders did not show any association with hospitalization due to fractures.

**Fig 2:**
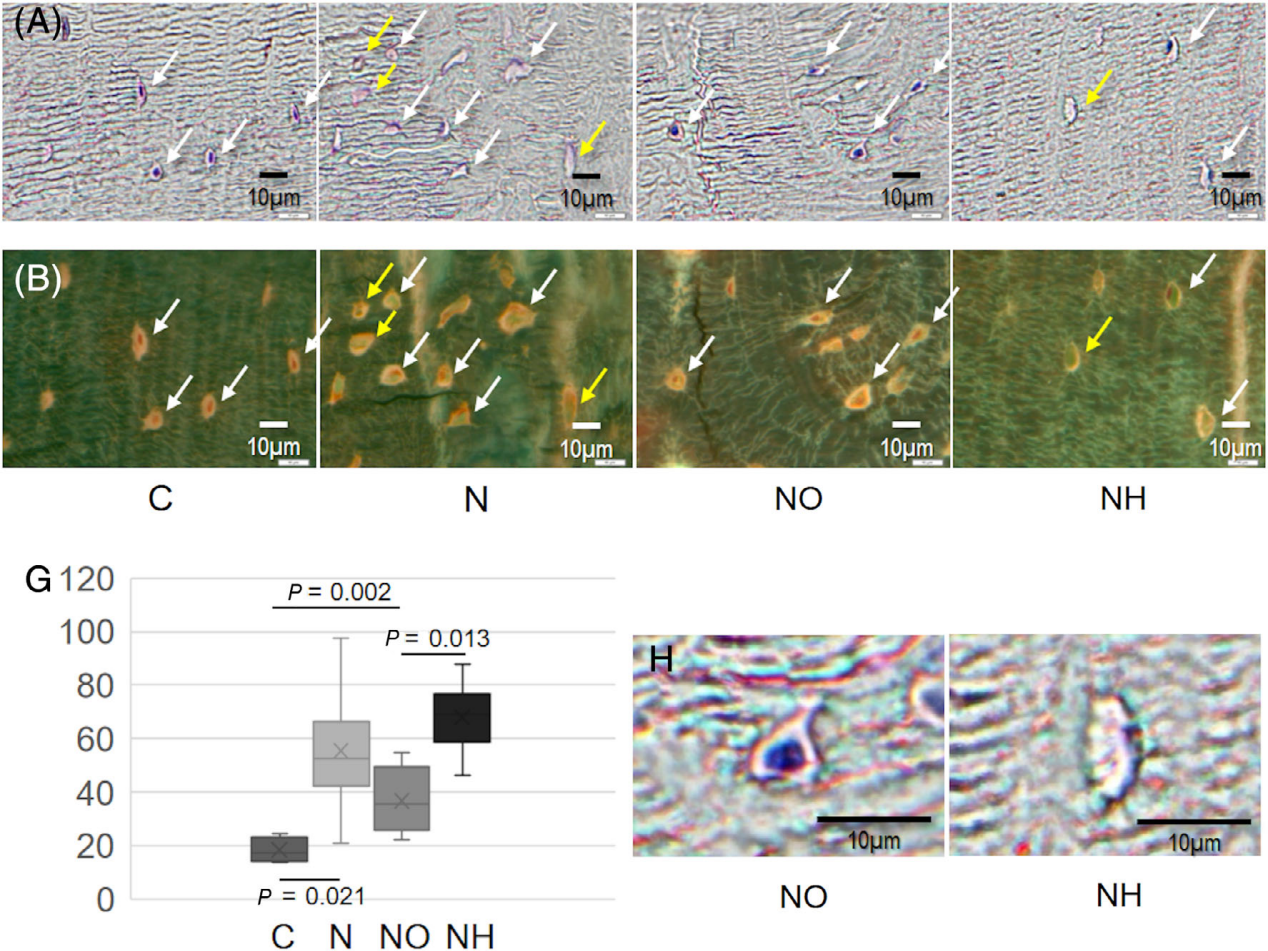
Hypothetical mechanism of bone fragility caused by decreased kidney function and the role of AT‐1RB.

### Effect of olmesartan use on kidney damage‐induced deterioration of bone material properties in vivo

Forty‐six animals (group C: 6, N: 14, NO: 13, NH: 13) were included in the experiment, and 17 (group C: 0, N: 6, NO: 4. NH: 7) died during the observation period because of uremia. The remaining 29 were subjected to the analyses. Results of the in vivo study are summarized in Table 2. Nephrectomized rats showed a significant loss of body weight, which was not improved by treatment with olmesartan or hydralazine. Significant hypertension was found in rats in group N; treatment with either olmesartan or hydralazine decreased the blood pressure to similar values to those in group C. In the biochemical analyses, azotemia and hyperparathyroidism was significantly increased in the nephrectomized groups and treatment with antihypertensive drugs showed no effect. Femoral bone density significantly decreased in nephrectomized rats, and treatment with antihypertensive drugs did not improve osteopenia(Table 2). Bone histomorphometric testing revealed an increased tendency in eroded surfaces and osteoclastic numbers that enhanced bone resorption and osteoblastic/osteoid surfaces that enhanced bone formation in nephrectomized groups. There was no difference in these parameters among the three nephrectomized groups.

**Table 2:** Demographic, Bone Histomorphometric, and Bone Mechanical Property in Rat Model

Group	C (*n* = 6)	N (*n* = 8)	NO (*n* = 9)	NH (*n* = 6)
Body weight (g)	647.0 ± 45.4	545.5 ± 67.4^a^	528.9 ± 48.6^a^	533.3 ± 43.7^a^
MBP (mmHg)	110.8 ± 13.5	148.9 ± 13.9^a^	114.9 ± 28.9^b^	116.8 ± 10.9^b^
Biochemistry				
Urea nitrogen (mg/dL)	30.6 ± 4.3	103.8 ± 24.7^a^	99.0 ± 55.3^a^	101.0 ± 25.6^a^
Creatinine (mg/dL)	0.5 ± 0.1	1.7 ± 0.3^a^	1.6 ± 0.8^a^	1.7 ± 0.8^a^
Calcium (mg/dL)	10.7 ± 1.6	11.1 ± 1.1	11.6 ± 0.9	11.5 ± 0.7
Phosphate (mg/dL)	7.8 ± 1.1	7.8 ± 1.4	7.9 ± 1.9	7.2 ± 2.0
Intact PTH (pg/dL)	109.5 ± 129.3	1141.6 ± 830.6	894.0 ± 429.5^a^	679.0 ± 291.9
BMD (mg/cm^3^)	177.5 ± 6.8	160.2 ± 6.6^a^	151.2 ± 15.6^a^	149.7 ± 12.1^a^
Bone histomorphometry				
ES/BS (%)	5.9 ± 2.1	10.7 ± 3.4^a^	9.8 ± 5.3^a^	10.8 ± 7.2^a^
N.Oc/B.Pm (/mm)	1.5 ± 0.6	2.0 ± 0.7	2.5 ± 0.8^a^	2.3 ± 0.5^a^
Ob.S/BS (%)	4.6 ± 2.7	14.1 ± 2.8^a^	16.5 ± 7.3^a^	13.7 ± 3.6^a^
OS/BS (%)	7.3 ± 4.0	26.8 ± 13.5^a^	29.6 ± 14.5^a^	30.7 ± 15.7^a^
Elastic bone mechanical property				
Storage module (Pa)	7.7 × 10^9^ ± 2.5 × 10^8^	2.5 × 10^9^ ± 7.9 × 10^8 a^	3.5 × 10^9^ ± 4.6 × 10^8 a,b,c^	1.5 × 10^9^ ± 8.3 × 10^8 a^
Tan delta	0.064 ± 0.0012	0.061 ± 0.017	0.060 ± 0.010	0.056 ± 0.021
Bone apatite orientation	6.9 ± 0.3	5.4 ± 0.6^a^	6.8 ± 0.2^b^ ^,^ ^c^	6.6 ± 0.3^a^

C = control group; N = nephrectomy group; NO = nephrectomy with olmesaltan group; NH = nephrectomy with hydralazine group; MBP = mean blood pressure; BMD = bone mineral density; BS = bone surface; ES = eroded surface; N.Oc = osteoclast number; Ob.S = osteoblastic surface; OS = osteoid surface.

^a^ *p* < 0.05 versus C.

^b^ *p* < 0.05 versus N.

^c^ *p* < 0.05 versus NH.

DMA of femurs showed that nephrectomized rats had significantly decreased storage module levels than those in group C (N: 2.5 × 10^9^ ± 7.9 × 10^8^ pa versus C: 7.7 × 10^9^ ± 2.5 × 10^8^ pa, *p* < 0.05). Treatment with olmesartan, but not with hydralazine, partly limited the deterioration (NO: 3.5 × 10^9^ ± 4.6 × 10^8^ pa versus NH: 1.5 × 10^9^ ± 8.3 × 10^8^ pa, *p* < 0.05) (Table 2). Nephrectomy caused an increase in the number of empty lacunae in the cortical bone (N: 55.3 ± 22.3/mm^2^ versus C: 18.3 ± 4.6/mm^2^, *p =* 0.021), whereas olmesartan use led to a decrease in the number of empty lacunae compared with hydralazine (NO: 36.6 ± 12.2/mm^2^ versus NH: 67.9 ± 13.6/mm^2^, *p =* 0.013) (Fig. 2). X‐ray diffraction study revealed significant bone apatite disorientation in nephrectomized rats (N: 5.4 ± 0.6 versus C: 6.9 ± 0.3, *p* < 0.001), whereas treatment with olmesartan treatment improved this value to a level comparable to that in control rats (NO: 6.8 ± 0.2 versus C: 6.9 ± 0.3, NS).

**Fig 3:**
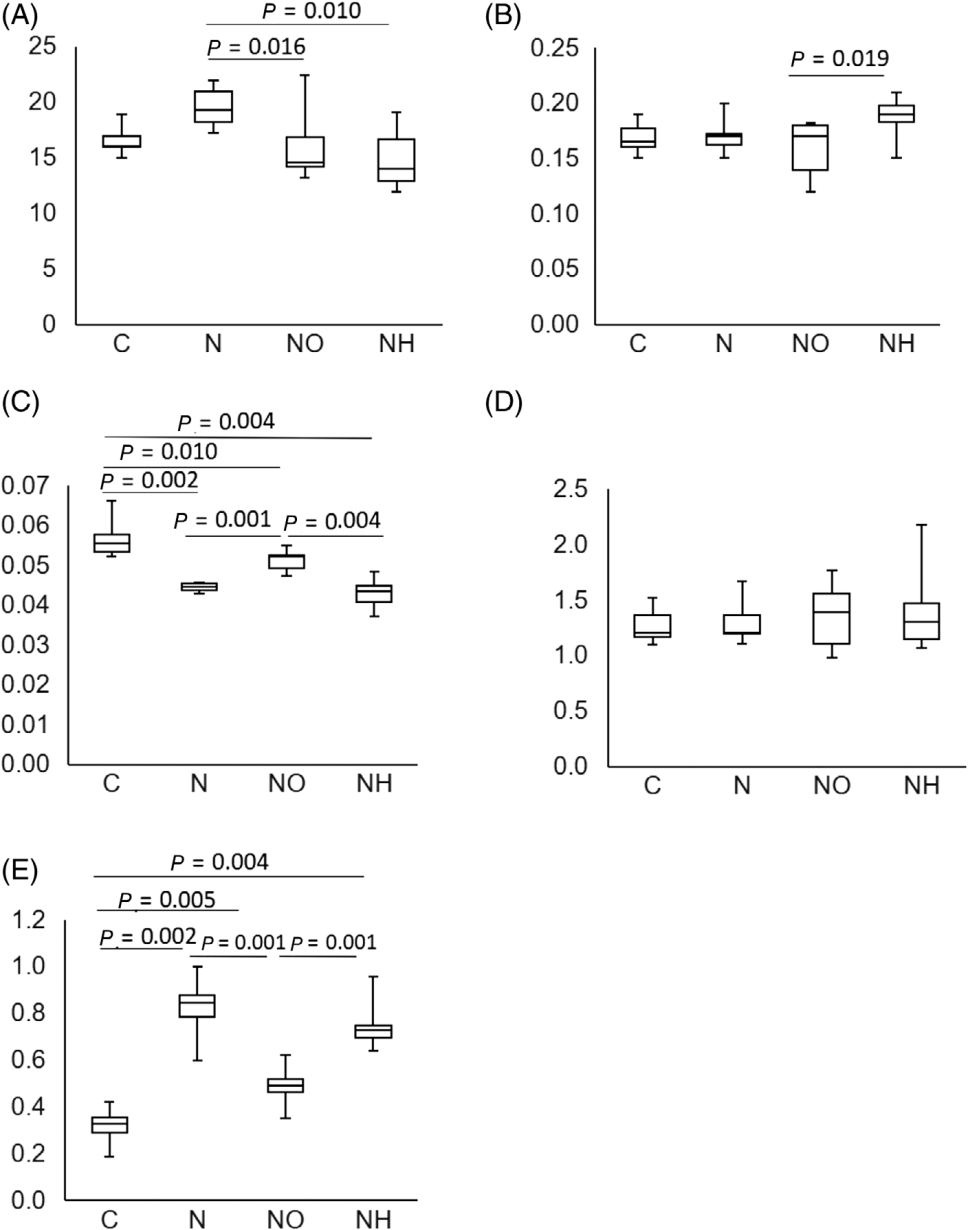
Improvement in the kidney damage‐induced increase of empty lacunae in cortical bone after olmesartan treatment. Bone histology showing the number of empty lacunae in cortical bone (N.Empty Lc/mm^2^) in a rat model. (A) Natural light microscopy findings. (*B*) fluorescence microscopy findings. C = control groups; N = nephrectomy groups; NO = nephrectomy with olmesartan groups; NH = nephrectomy with hydralazine groups. White arrows show intact osteocytes, and yellow arrows show empty lacunae. Scale bars = 10 mm. G = Quantitative comparison of the number of empty lacunae among groups.

Results of Raman‐spectroscopic analyses are shown in Fig. 3. Crystallinity was significantly decreased in nephrectomized rats, which was partially improved by olmesartan treatment (NO: 0.051 ± 0.001 versus N: 0.044 ± 0.001, *p =* 0.001). Pentosidine was significantly accumulated in nephrectomized rats, but this effect was countered by treatment with olmesartan. (NO: 0.49 ± 0.08 versus N: 0.81 ± 0.14, *p =* 0.001).

**Fig 4:**
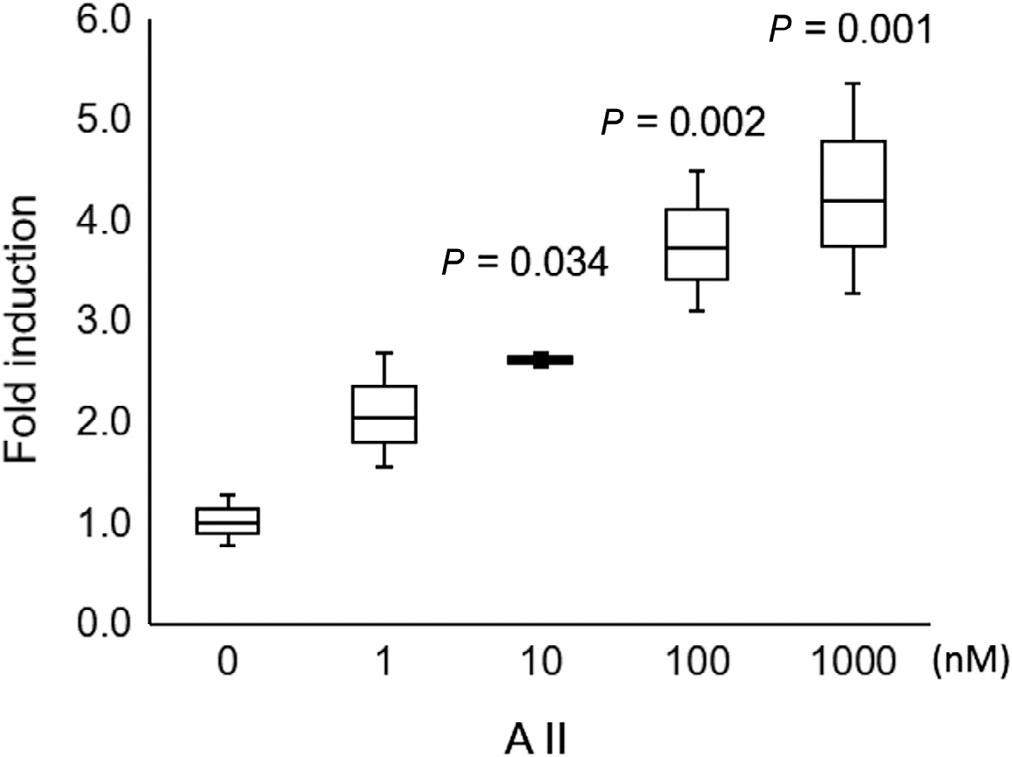
Effect of olmesartan on nephrectomy‐induced bone chemical composition in a rat model. (*A*) Mineral‐to‐matrix ratio; (*B*) carbonate‐to‐phosphate ratio; (*C*) crystallinity; (*D*) cross‐links; and (*E*) pentosidine‐to‐matrix ratio in C (control groups), N (nephrectomy groups), NO (nephrectomy with olmesartan groups), and NH (nephrectomy with hydralazine groups) are shown.

### Effect of olmesartan use on angiotensin II‐induced abnormal osteocyte functions in vitro

When primary cultured osteocytes reacted with angiotensin II in vitro, the angiotensin II increased *AT‐1R* mRNA expression (Fig. 4), decreased cell viability after 96 hours (73.3 ± 2.8% of control, *p* < 0.001) (Fig. 5), and increased the ROS production (148.2 ± 19.4% of control, *p =* 0.004) (Fig. 6). Olmesartan, but not hydralazine, ameliorated the angiotensin II‐induced impairment of cell viability and ROS production (cell viability: 93.4 ± 8.3% of control, *p =* 0.011; ROS production: 114.5 ± 17.4% of control, *p =* 0.003) (Fig. 7*A*, *B*). Angiotensin II increased DNA fragmentation, NADPH oxidase, p22phox, and p67phox mRNA expressions in osteocytes, whereas the opposite effect was found after olmesartan treatment (Fig. 7*C–E*).

**Fig 5:**
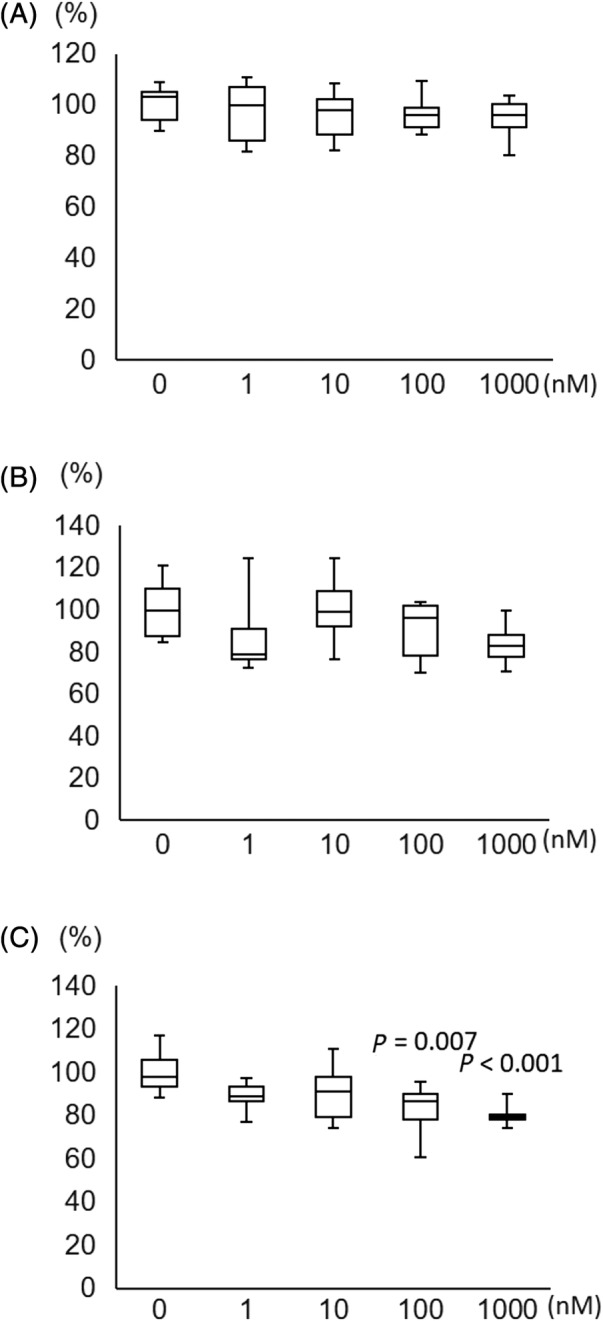
Expression of type I angiotensin II receptor 1 (AT‐1R) in primary cultured osteocytes. Cells were treated with different concentrations of angiotensin II for 24 hours. AT‐1R expression was assayed by real‐time reverse transcription PCR. Fold induction of AT‐1R gene expression was compared with beta‐actin. Data are obtained from three independent experiments. *p* value versus 0 mM AII.

**Fig 5:**
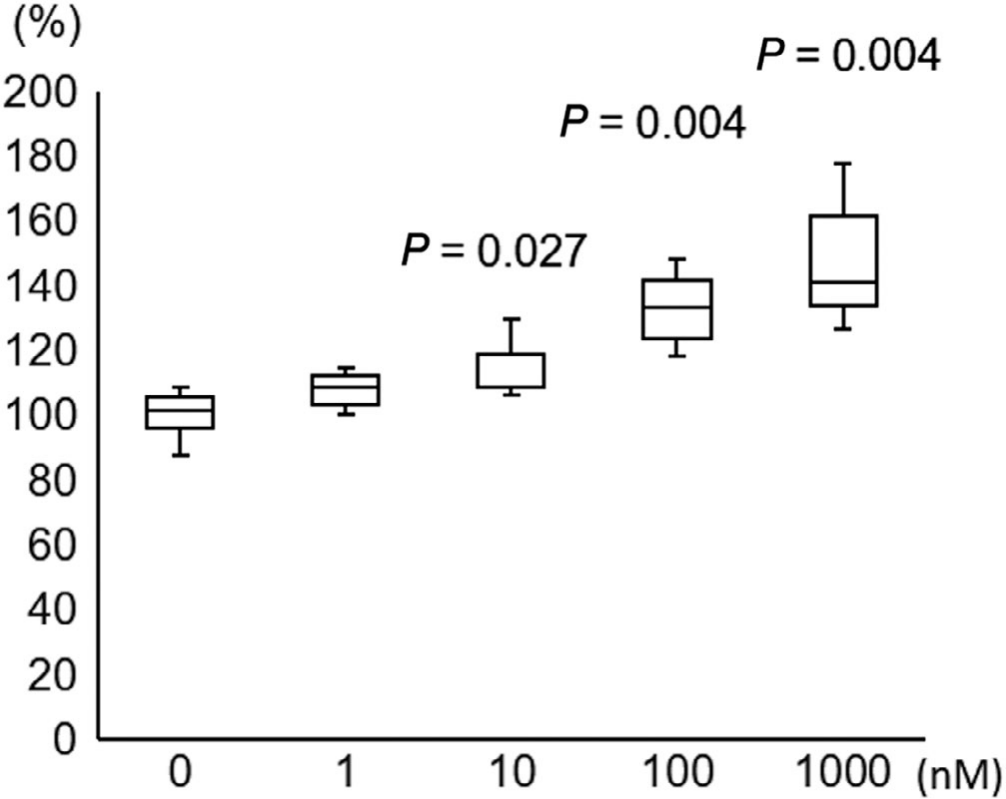
Effect of angiotensin II on the viability of primary osteocytes. Primary cultured osteocytes were exposed to several concentrations of angiotensin II (AII) for 24 hours (*A*), 48 hours (*B*), and 96 hours (*C*). Cell viability decreased in a dose‐dependent manner after a 96‐hour exposure to AII. *p* value versus 0 mM AII.

**Fig 7:**
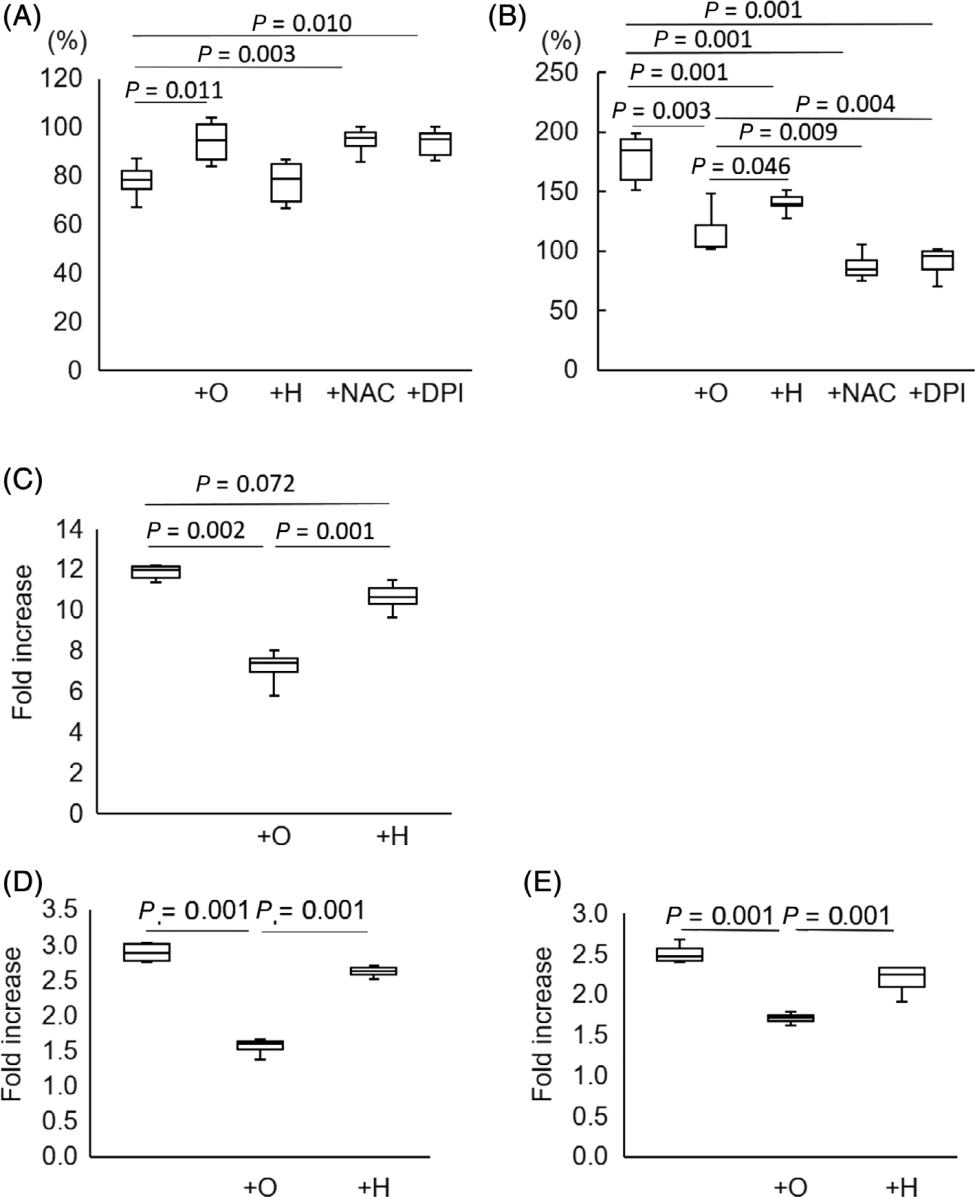
Effect of angiotensin II on reactive oxygen spices (ROS) production in primary osteocytes. Angiotensin II induced ROS production in primary osteocytes in a dose‐dependent manner. *p* value versus 0 mM AII.

**Fig 8:**
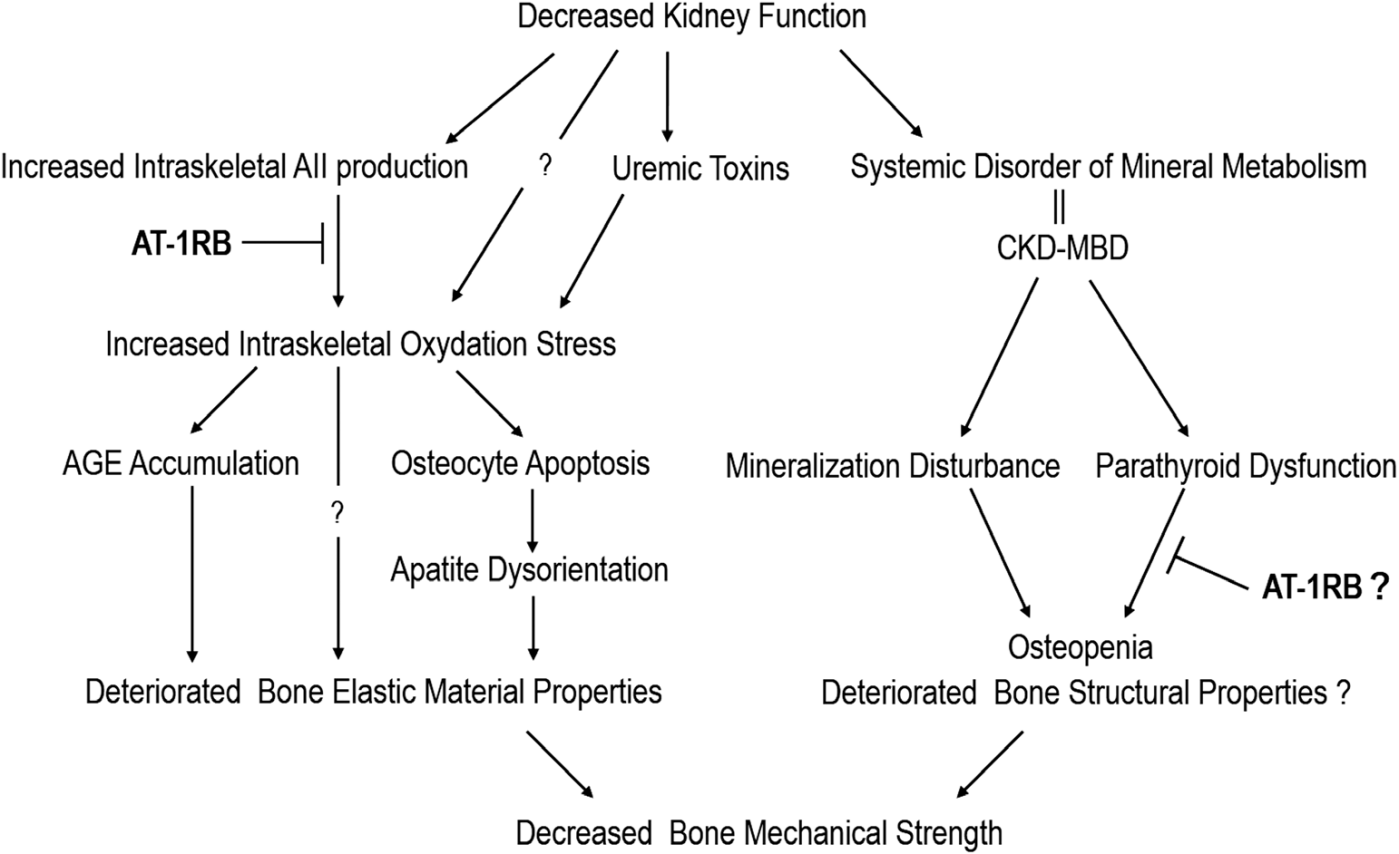
Improvement in angiotensin II‐induced cell death and ROS production in osteocytes after olmesartan treatment. Primary cultured osoteocytes in the presence of 1000 μM angiotensin II reacted with 10 μM olmesartan (+O), 30 μM hydralazine (+H), 2.5 mM N‐acetyl cysteine (+NAC), 0.5 μM diphenyleneiodonium (+DPI), or without drugs (C). Cell viability (*A*), ROS production (*B*), DNA fragmentation (*C*), and NADPH oxidase expression including p22phox (*D*) and p67phox (*E*) were assessed, respectively. All data were compared with that of angiotensin II non‐treated cells and expressed as fold changes or increase. Data are presented as mean + standard deviation of three independent experiments. *p* value versus C.

## Discussion

Previous clinical studies have suggested that the relationship between fracture risk and PTH levels in patients with CKD patients is unclear.^(^ ^2^, ^3^, ^4^, ^5^, ^6^, ^7^ ^)^ Our study also showed that three CKD‐MBD markers, including serum levels of PTH, calcium, and phosphorus, were not associated with fracture incidence in patients undergoing hemodialysis (Table 1 and Fig. 1). Although recent advancements in treatment with activated vitamin D, calcium‐sensing receptor agonists, and phosphate binders have contributed to maintaining the circulation levels of the above three CKD markers,^(^ ^21^ ^)^ the risk of fracture in these patients remains much higher than that in the general population.^(^ ^8^, ^9^, ^10^, ^11^, ^12^ ^)^

We focused on the effect of AT‐1RB use on hospitalization due to fractures and bone quality in clinical and basic experiments. This clinical observational study revealed that the administration of AT‐1RB but not calcium channel blockers was associated with a lower fracture incidence among patients undergoing hemodialysis (Fig. 1). Compared with that in a previous study,^(^ ^19^ ^)^ the effect of AT‐1RB seemed to be stronger in patients undergoing hemodialysis than in the general population. In an animal model of experimentally induced kidney injury, intraskeletal protein expression of angiotensin II was significantly increased.^(^ ^29^ ^)^ If this phenomenon is reproducible in patients with CKD, it may account for the osteoprotective effect of AT‐1RB use noted in our study. Hypovitaminosis D is a possible cause of high AII levels in chronic kidney injury via the activation of renin gene expression and subsequent activation of the renin‐angiotensin‐aldosterone system.^(^ ^30^ ^)^ Elevated AII levels downregulate renal klotho expression through an AT‐1R‐dependent manner.^(^ ^31^ ^)^

Recent observational studies showed that incidence of hip fractures in patients undergoing hemodialysis is decreasing,^(^ ^32^, ^33^ ^)^ which may be due to the increasing use of AT‐1RB. However, the pharmacological mechanisms of fragility fracture prevention through AT‐1RB use are not known to date. We examined the effect of AT‐1RB use on kidney damage‐induced bone abnormalities separately from their suppressive effect on osteoclastogenesis.

In animals with experimentally induced kidney injury, although the femoral bone elastic mechanical properties and chemical compositions were shown to be impaired, the administration of olmesartan partially restored these parameters independently of changes in the blood pressure (Table 2 and Fig. 2). In contrast, olmesartan did not improve the decrease in bone mass noted in these animals, indicating that the improvement in bone elastic mechanical properties through AT‐1RB use is not associated with changes in the bone mass. Although a previous study suggested that the administration of olmesartan was associated with mild suppression of osteoclastic bone resorption,^(^ ^17^ ^)^ no significant suppressive effect of olmesartan on bone resorption was demonstrated in our study. We do not fully exclude the possibility that olmesartan suppresses bone resorption from this result, but it was at least not sufficient to reduce bone mass in this experimental setting. In contrast, olmesartan restored the uremia‐induced increase in the pentosidine/amide ratio and apatite disorientation, which could contribute to the partial storage modulus increase without the increase in the bone mass. Similar to that noted in previous studies,^(^ ^13^, ^14^, ^15^ ^)^ the inhibition of these two pathological reactions was due to the direct interaction between the bone and olmesartan. The increased number of empty lacunae observed after kidney damage was also improved on olmesartan administration (Fig. 2). An increased number of empty lacunae suggests the promotion of osteocyte death. Thus, the inhibition of the AT‐1R with olmesartan partially improved the deterioration of bone elastic mechanical properties without changes in the bone mass in CKD animals.

In the in vitro study, the exposure to angiotensin II induced DNA fragmentation, which led to increased osteocyte apoptosis (Fig. 7*C*). Olmesartan inhibited angiotensin II‐induced osteocytic functional abnormalities (Fig. 7). Angiotensin II is one of the strongest local oxidation stress factors.^(^ ^34^ ^)^ Thus, the intraskeletal oxidation stress induced by angiotensin II is one of the likely causes of osteocyte death noted in rats with kidney injury (Fig. 8). Although the mechanism underlying establishment of apatite orientation is not known to date, it is known that the orientation relies on in vivo stress distribution in weight‐bearing bones, while the orientation determinants in non‐weight‐bearing bones have not been identified to date.^(^ ^35^ ^)^ The skeletal load sensor is the osteocyte network. Therefore, a blockade of the osteocyte network by osteocyte apoptosis would dissolve the association between the direction of in vivo stress and apatite orientation, by which bones accommodate the stress imposed upon them. We consider that this is the pathophysiological mechanism by which apatite orientation in weight‐bearing bones is affected in patients with CKD. We detected a protective effect of olmesartan on this orientation through the inhibition of osteocyte apoptosis. Previous studies have reported that apatite orientation is affected by the increased osteocyte death observed in osteoporosis due to calcium depletion or abnormalities in the osteocyte network arrangement noted in melanoma bone metastasis, which supports the present assumption.^(^ ^36^ ^)^ The intraskeletal oxidation stress caused by local angiotensin II production may have also contributed toward the accumulation of advanced glycation end products as a non‐physiological type I collagen cross‐link in chronic kidney injury, which is another causative factor for the deterioration in bone material properties.^(^ ^37^ ^)^

In summary, we demonstrate that AT‐1RB contributes to the underlying pathogenesis of abnormal bone quality in the setting of CKD, possibly by reducing oxidative stress. However, AT‐1RB use did not lead to a complete recovery of deteriorated bone elastic mechanical properties or of the level of osteocyte apoptosis. We previously demonstrated that uremic toxins that could be absorbed by oral charcoal absorbents restored bone elastic properties.^(^ ^14^ ^)^ Angiotensin II would, therefore, be one of those uremic toxins that deteriorate bone elastic mechanical property through promoting local oxidation stress (Fig. 6). Recently, it was reported that reduction of total bone AGE using the AGE cross‐link breaker failed to improve bone mechanical properties in animals with chronic kidney injury.^(^ ^38^ ^)^ We assume they failed because AGE accumulation is not the only pathophysiological mechanism underlying deteriorated bone elastic mechanical properties associated with chronic kidney injury, as this study demonstrated.

There are several limitations of this study. First, a lack of data on bone mass is a limitation of this clinical study. In the animal study, we provided a high‐calcium diet to limit the possible effect of PTH on the study results, while significant secondary hyperparathyroidism was still detected in the nephrectomized groups. A previous study reported that parathyroid function was not a factor affecting bone elastic material properties;^(^ ^14^ ^)^ our study also showed that olmesartan did not affect PTH levels in nephrectomized rats. Even considering the aforementioned limitations, we believe that the study findings are significant.

We found that bone fragility in uremic conditions is not completely caused by a systemic disorder of mineral metabolism. CKD‐MBD may not always be the cause of CKD‐related bone fragility. Osteoporosis is a skeletal disorder characterized by compromised bone strength, predisposing the patients to an increased risk of fracture,^(^ ^39^ ^)^ regardless of its cause. Reduced bone mass is not a requirement. Therefore, we should consider the cause of fragility fracture in patients with CKD to be osteoporosis, which is possibly caused by multiple factors, such as primary osteopenia, CKD‐MBD, and intraskeletal oxidation stress.^(^ ^40^, ^41^, ^42^ ^)^ This study revealed that the administration of AT‐1RB is a promising treatment option against intraskeletal oxidation stress and that it partially improves the abnormalities in bone elastic mechanical properties in CKD. Moreover, increased angiotensin II production and intraskeletal oxidation stress was also found in an aged animal model.^(^ ^43^ ^)^ Administration of AT‐1RB may be a useful treatment strategy for elderly patients with osteoporosis, and further studies are, therefore, warranted on the topic.

## Disclosures

The MBD‐5D study was financially supported by Kyowa Kirin Co., Ltd. (KK). SY has received honoraria from KK. SF has acted as a scientific advisor for KK. TA has received consulting fees from KK, Astellas Pharma, Bayer, Fuso Pharmaceutical, Japan Tobacco, Ono Pharmaceutical, Otsuka, Sanwa, GSK, and NIPRO and lecture fees from KK, Chugai Pharmaceutical, Bayer, Kissei Pharmaceutical, Japan Tobacco, and Ono Pharmaceutical. SF has acted as a scientific advisor for and has received grants from KK. MF has received consulting fees from KK and Ono Pharmaceutical; lecture fees from KK, Bayer, Kissei Pharmaceutical, Japan Tobacco, and OnoPharmaceutical; and grants from KK and Bayer. JJK has received lecture fees from KK, Japan Tobacco, Chugai Pharmaceutical, Kissei Pharmaceutical, and Ono Pharmaceutical. All other authors state that they have no conflicts of interest.

### Peer Review

The peer review history for this article is available at https://publons.com/publon/10.1002/jbmr.4159.

## Supporting information


Supplemental Table S1.

## References

[1] Moe SM , Drueke T , Lameire N , Eknoyan G . Chronic kidney disease‐mineral‐bone disorder: a new paradigm. Adv Chronic Kidney Dis. 2007;14:3–12. 17200038 10.1053/j.ackd.2006.10.005

[2] Coco M , Rush H . Increased incidence of hip fractures in dialysis patients with low serum parathyroid hormone. Am J Kidney Dis. 2000;36:1115–21. 11096034 10.1053/ajkd.2000.19812

[3] Jadoul M , Albert JM , Akiba T , et al. Incidence and risk factors for hip or other bone fractures among hemodialysis patients in the dialysis outcomes and practice patterns study. Kidney Int. 2006;70:1358–66. 16929251 10.1038/sj.ki.5001754

[4] Ambrus C , Almasi C , Berta K , et al. Vitamin D insufficiency and bone fractures in patients on maintenance hemodialysis. Int Urol Nephrol. 2011;43:475–82. 20237846 10.1007/s11255-010-9723-x

[5] Atsumi K , Kushida K , Yamazaki K , Shimizu S , Ohmura A , Inoue T . Risk factors for vertebral fractures in renal osteodystrophy. Am J Kidney Dis. 1999;33:287–93. 10023640 10.1016/s0272-6386(99)70302-1

[6] Stehman‐Breen CO , Sherrard DJ , Alem AM , et al. Risk factors for hip fracture among patients with end‐stage renal disease. Kidney Int. 2000;58:2200–5. 11044242 10.1111/j.1523-1755.2000.00394.x

[7] Danese MD , Kim J , Doan QV , Dylan M , Griffiths R , Chertow GM . PTH and the risks for hip, vertebral, and pelvic fractures among patients on dialysis. Am J Kidney Dis. 2006;47:149–56. 16377396 10.1053/j.ajkd.2005.09.024

[8] Alem A , Sherrard D , Gillen D , et al. Increased risk of hip fracture among patients with end‐stage renal disease. Kidney Int. 2000;58:396–9. 10886587 10.1046/j.1523-1755.2000.00178.x

[9] Wakasugi M , Kazama JJ , Taniguchi M , et al. Increased risk of hip fracture among Japanese hemodialysis patients. J Bone Miner Metab. 2013;31:315–21. 23292163 10.1007/s00774-012-0411-z

[10] Tentori F , McCullough K , Kilpatrick RD , et al. High rates of death and hospitalization follow bone fracture among hemodialysis patients. Kidney Int. 2014;85:166–73. 23903367 10.1038/ki.2013.279 PMC3910091

[11] Maravic M , Ostertag A , Urena P , Cohen‐Solal M . Dementia is a major risk factor for hip fractures in patients with chronic kidney disease. Osteoporosis Int. 2016;27:1665–9. 10.1007/s00198-015-3429-y 26588907

[12] Hansen D , Olesen JB , Gislason GH , Abrahamsen B , Hommel K . Risk of fracture in adults on renal replacement therapy: a Danish national cohort study. Nephrol Dialysis Transp. 2016;31:1654–62. 10.1093/ndt/gfw073 27190324

[13] Iwasaki Y , Kazama JJ , Yamato H , Shimoda H , Fukagawa M . Accumulated uremic toxins attenuate bone mechanical properties in rats with chronic kidney disease. Bone. 2013;57:477–83. 23920356 10.1016/j.bone.2013.07.037

[14] Iwasaki Y , Kazama JJ , Yamato H , Matsugaki A , Nakano T , Fukagawa M . Altered material properties are responsible for bone fragility in rats with chronic kidney injury. Bone. 2015;81:247–54. 26187196 10.1016/j.bone.2015.07.015

[15] Iwasaki Y , Kazama JJ , Yamato H , Fukagawa M . Changes in chemical composition of cortical bone associated with bone fragility in rat model with chronic kidney disease. Bone. 2011;48:1260–7. 21397740 10.1016/j.bone.2011.03.672

[16] Bandow K , Nishikawa Y , Ohnishi T , et al. Low‐intensity pulsed ultrasound (LIPUS) induces RANKL, MCP‐1, and MIP‐1beta expression in osteoblasts through the angiotensin II type 1 receptor. J Cell Physiol. 2007;211:392–8. 17167786 10.1002/jcp.20944

[17] Shimizu H , Nakagami H , Osako MK , et al. Angiotensin II accelerates osteoporosis by activating osteoclasts. FASEB J. 2008;22:2465–75. 18256306 10.1096/fj.07-098954

[18] Yamamoto S , Kido R , Onishi Y , et al. Use of renin‐angiotensin system inhibitors is associated with reduction of fracture risk in hemodialysis patients. PLoS One. 2015;10:e0122691. 25874620 10.1371/journal.pone.0122691 PMC4395204

[19] Rejnmark L , Vestergaard P , Mosekilde L . Treatment with beta‐blockers, ACE inhibitors, and calcium‐channel blockers is associated with a reduced fracture risk: a nationwide case‐control study. J Hypertens. 2006;24:581–9. 16467662 10.1097/01.hjh.0000203845.26690.cb

[20] Fukuhara S , Akizawa T , Fukagawa M , et al. Mineral and bone disorders outcomes study for Japanese chronic kidney disease stage 5D patients: rationale and study design. Ther Apher Dial. 2011;15:169–75. 21426510 10.1111/j.1744-9987.2010.00906.x

[21] Fukagawa M , Yokoyama K , Koiwa F , et al. Clinical practice guideline for the management of chronic kidney disease‐mineral and bone disorder. Ther Apher Dial. 2013;17:247–88. 23735142 10.1111/1744-9987.12058

[22] Kubota Y , Umegaki K , Kagota S , et al. Evaluation of blood pressure measured by tail‐cuff methods (without heating) in spontaneously hypertensive rats. Biol Pharm Bull. 2006;29:1756–8. 16880638 10.1248/bpb.29.1756

[23] Dempster DW , Compston JE , Drezner MK , et al. Standardized nomenclature, symbols, and units for bone histomorphometry: a 2012 update of the report of the ASBMR Histomorphometry Nomenclature Committee. J Bone Miner Res. 2013;28:2–17. 23197339 10.1002/jbmr.1805 PMC3672237

[24] Mikuni‐Takagaki Y , Kakai Y , Satoyoshi M , et al. Matrix mineralization and the differentiation of osteocyte‐like cells in culture. J Bone Miner Res. 1995;10:231–42. 7754802 10.1002/jbmr.5650100209

[25] Stern AR , Stern MM , Van Dyke ME , Jahn K , Prideaux M , Bonewald LF . Isolation and culture of primary osteocytes from the long bones of skeletally mature and aged mice. Biotechniques. 2012;52:361–73. 22668415 10.2144/0000113876 PMC3612989

[26] Nii‐Kono T , Iwasaki Y , Uchida M , et al. Indoxyl sulfate induces skeletal resistance to parathyroid hormone in cultured osteoblastic cells. Kidney Int. 2007;71:738–43. 17264878 10.1038/sj.ki.5002097

[27] Motojima M , Hosokawa A , Yamato H , Muraki T , Yoshioka T . Uremic toxins of organic anions up‐regulate PAI‐1 expression by induction of NF‐kappaB and free radical in proximal tubular cells. Kidney Int. 2003;63:1671–80. 12675842 10.1046/j.1523-1755.2003.00906.x

[28] Motojima M , Hosokawa A , Yamato H , Muraki T , Yoshioka T . Uraemic toxins induce proximal tubular injury via organic anion transporter 1‐mediated uptake. Br J Pharmacol. 2002;135:555–63. 11815391 10.1038/sj.bjp.0704482 PMC1573145

[29] Gu SS , Zhang Y , Wu SY , Diao TY , Gebru YA , Deng HW . Early molecular responses of bone to obstructive nephropathy induced by unilateral ureteral obstruction in mice. Nephrology (Carlton). 2012;17:767–73. 22943310 10.1111/j.1440-1797.2012.01656.x

[30] Li YC , Kong J , Wei M , Chen ZF , Liu SQ , Cao LP . 1,25‐dihydroxyvitamin D(3) is a negative endocrine regulator of the renin‐angiotensin system. J Clin Invest. 2002;110:229–38. 12122115 10.1172/JCI15219 PMC151055

[31] Mitani H , Ishizaka N , Aizawa T , et al. In vivo klotho gene transfer ameliorates angiotensin II‐induced renal damage. Hypertension. 2002;39:838–43. 11967236 10.1161/01.hyp.0000013734.33441.ea

[32] Arneson TJ , Li S , Liu J , Kilpatrick RD , Newsome BB , St Peter WL . Trends in hip fracture rates in US hemodialysis patients, 1993‐2010. Am J Kidney Dis. 2013;62:747–54. 23631997 10.1053/j.ajkd.2013.02.368

[33] Wakasugi M , Kazama JJ , Wada A , Hamano T , Masakane I , Narita I . Hip fracture trends in Japanese dialysis patients, 2008‐2013. Am J Kidney Dis. 2018;71:173–81. 29162337 10.1053/j.ajkd.2017.07.017

[34] Hanna IR , Taniyama Y , Szöcs K , Rocic P , Griendling KK . NAD(P)H oxidase‐derived reactive oxygen species as mediators of angiotensin II signaling. Antioxid Redox Signal. 2002;4:899–914. 12573139 10.1089/152308602762197443

[35] van Oers RF , Wang H , Bacabac RG . Osteocyte shape and mechanical loading. Curr Osteoporos Rep. 2015;13:61–6. 25663071 10.1007/s11914-015-0256-1 PMC4352610

[36] Kimura Y , Matsugaki A , Sekita A , Nakano T . Alteration of osteoblast arrangement via direct attack by cancer cells: new insights into bone metastasis. Sci Rep. 2017;7:44824. 28303941 10.1038/srep44824 PMC5356003

[37] Saito M , Marumo K . Effects of collagen crosslinking on bone material properties in health and disease. Calcif Tissue Int. 2015;97:242–61. 25791570 10.1007/s00223-015-9985-5

[38] Chen NX , Srinivasan S , O'Neill K , et al. Effect of advanced glycation end‐products (AGE) lowering drug ALT‐711 on biochemical, vascular, and bone parameters in a rat model of CKD‐MBD. J Bone Miner Res. 2020;35:608–17. 31743501 10.1002/jbmr.3925 PMC9030558

[39] NIH Consensus Development Panel on Osteoporosis Prevention, Diagnosis, and Therapy . Osteoporosis prevention, diagnosis, and therapy. JAMA. 2001;285:785–95. 11176917 10.1001/jama.285.6.785

[40] Kazama JJ , Iwasaki Y , Fukagawa M . Uremic osteoporosis. Kid Int. 2013;3:446–50. 10.1038/kisup.2013.93 PMC4089591 25019028

[41] Kazama JJ . Chronic kidney disease and fragility fracture. Clin Exp Nephrol. 2017;21(Suppl 1):46–52. 28012057 10.1007/s10157-016-1368-3 PMC5306431

[42] Moe SM . Renal osteodystrophy or kidney‐induced osteoporosis? Curr Osteoporos Rep. 2017;15:194–7. 28497212 10.1007/s11914-017-0364-1 PMC5506492

[43] Gu SS , Zhang Y , Li XL , et al. Involvement of the skeletal renin‐angiotensin system in age‐related osteoporosis of ageing mice. Biosci Biotechnol Biochem. 2012;76:1367–71. 22785482 10.1271/bbb.120123

