# Supplementary figures and images for: Type I Angiotensin II Receptor Blockade Reduces Uremia‐Induced Deterioration of Bone Material Properties
Source: J Bone Miner Res. 2020 Oct 2;36(1):67–79. doi: 10.1002/jbmr.4159 (PMC9328427; doi:10.1002/jbmr.4159)

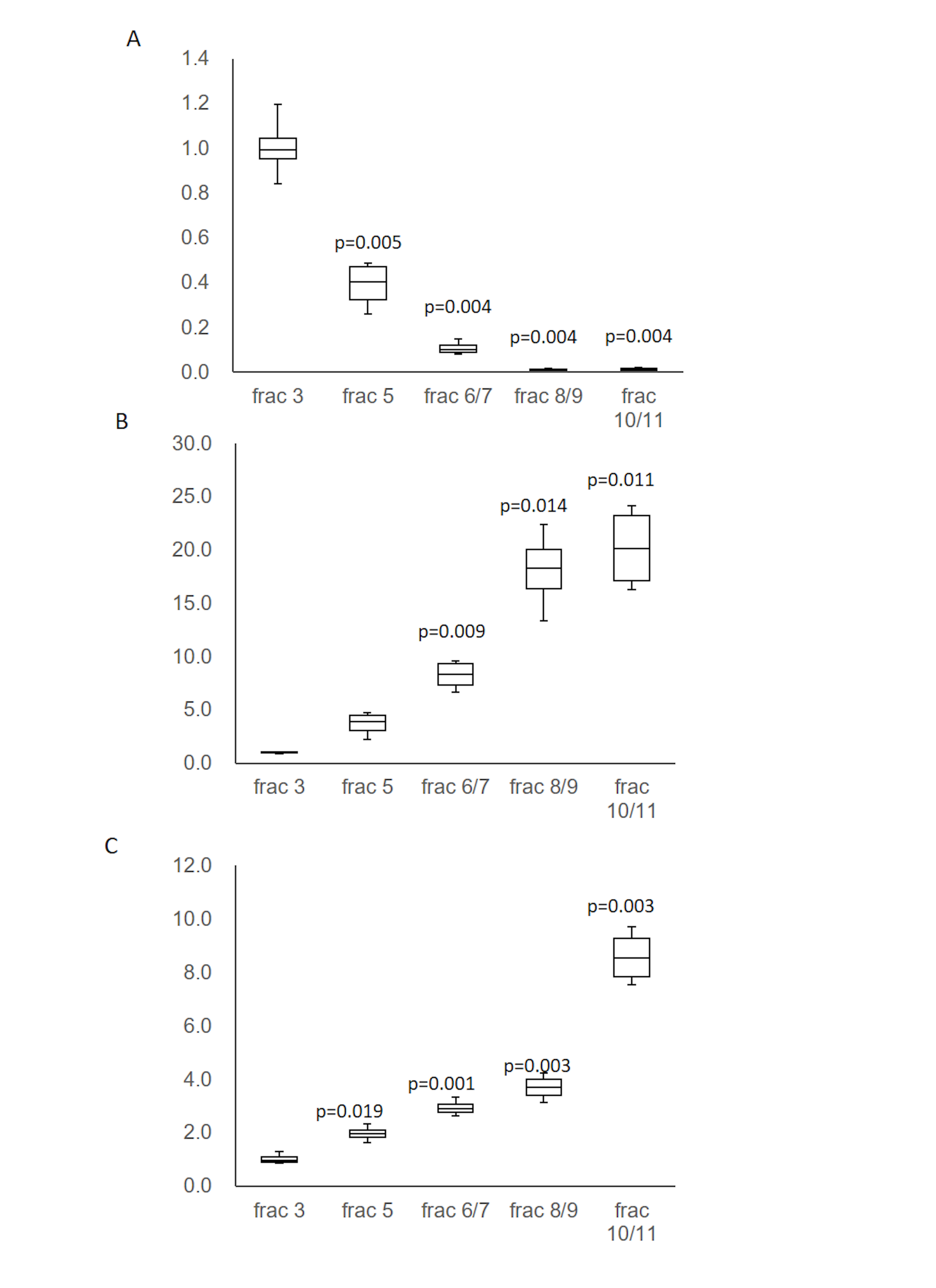

Supplement: Supplementary file 1 — Supplemental Table S1. [file JBMR-36-67-s001.tiff]
